# Crohn's Disease and Early Exposure to Domestic Refrigeration

**DOI:** 10.1371/journal.pone.0004288

**Published:** 2009-01-29

**Authors:** Fatemeh Malekzadeh, Corinne Alberti, Mehdi Nouraei, Homayoon Vahedi, Isabelle Zaccaria, Ulrich Meinzer, Siavosh Nasseri-Moghaddam, Rasoul Sotoudehmanesh, Sara Momenzadeh, Reza Khaleghnejad, Shahrooz Rashtak, Golrokh Olfati, Reza Malekzadeh, Jean-Pierre Hugot

**Affiliations:** 1 Digestive Disease Research Center, Medical Sciences, University of Tehran, Tehran, Iran; 2 APHP, Unité d'épidémiologie clinique, Hôpital Robert Debré, Paris, France; 3 INSERM CIE5, Paris, France; 4 AP-HP, service de gastroentérologie pédiatrique, Hôpital Robert Debré, Paris, France; 5 Université Paris Diderot, Paris, France; 6 INSERM U843, Paris, France; German Cochrane Center, Germany

## Abstract

**Background:**

Environmental risk factors playing a causative role in Crohn's Disease (CD) remain largely unknown. Recently, it has been suggested that refrigerated food could be involved in disease development. We thus conducted a pilot case control study to explore the association of CD with the exposure to domestic refrigeration in childhood.

**Methodology/Principal Findings:**

Using a standard questionnaire we interviewed 199 CD cases and 207 age-matched patients with irritable bowel syndrome (IBS) as controls. Cases and controls were followed by the same gastroenterologists of tertiary referral clinics in Tehran, Iran. The questionnaire focused on the date of the first acquisition of home refrigerator and freezer. Data were analysed by a multivariate logistic model. The current age was in average 34 years in CD cases and the percentage of females in the case and control groups were respectively 48.3% and 63.7%. Patients were exposed earlier than controls to the refrigerator (X2 = 9.9, df = 3, P = 0.04) and refrigerator exposure at birth was found to be a risk factor for CD (OR = 2.08 (95% CI: 1.01–4.29), P = 0.05). Comparable results were obtained looking for the exposure to freezer at home. Finally, among the other recorded items reflecting the hygiene and comfort at home, we also found personal television, car and washing machine associated with CD.

**Conclusion:**

This study supports the opinion that CD is associated with exposure to domestic refrigeration, among other household factors, during childhood.

## Introduction

Crohn's Disease (CD) is characterised by a chronic or relapsing inflammation of the digestive tract. The inflammation is supposed to occur in genetically at risk people when exposed to environmental risk factors. Genetic factors are now better known since several susceptibility genes or anonymous loci have been identified including CARD15/NOD2, 5q31 and 5p13 loci, TNFSF15, ATG16L1, IL23R, IRGM and others [1]–[6]. In contrast, the environmental risk factors are less well recognised except cigarette smoking which has been associated with an increased risk of the disease and with a more severe phenotype.

CD is frequent in industrialized areas such as Europe and North America, whereas it is rare in underdeveloped countries [7]. In countries where several ethnic groups coexist, those with a less westernised way of life have a lower risk for developing the disease. This was the case for example for the indigenous populations in South Africa or the Maoris in New Zealand [8], [9] when contrasted with European settlers. These variations have been explained by both genetic predisposition and environmental variations.

A long-term apparent increase in the incidence of CD is widely observed [10]–[12]. Uncontrolled biases such as case identification have previously been discussed and may contribute to this observation. However most of the authors usually agree with the reality of an outbreak of CD during the second part of the twentieth century. In westernised countries, the starting date of this outbreak is difficult to define precisely but population-based data suggest that CD incidence increased in the 40's or before in USA [12], in the 50's or before in Sweden [13], [14], in the 60's in United Kingdom [10], [11], [15], [16], and only later in Southern Europe. These different starting dates may in part explain the North-South gradient of incidence observed in Europe [17].

The long-term increase of the incidence indicates that environmental factors play a role in CD. For a complex genetic trait like CD, it can thus be postulated that genetically predisposed people were progressively recruited with the generalisation of the exposure to risk factors, explaining the outbreak. This point of view is consistent with the progressive standardisation of the modern life-style in developed countries during the second half of the 20^th^ century. Furthermore the relative stabilisation of the disease incidence in North America and in some European countries [18]–[20] suggests that the exposure to the risk factor is now widespread in these countries and that a vast majority of genetically at-risk people are now affected by the disease. However, it is to note that increasing incidences have also been reported recently in Scandinavia [21], [22].

Many crucial changes occurred during the 20^th^ century in food, housing, travels, leisure, clothes, health, etc, suggesting many candidate risk factors for CD [23]. Furthermore, all these parameters are interrelated and it is difficult to specifically extract the relevant one. However, it is to note that an association of CD with good standards of domestic hygiene in childhood (such as hot running water) has been reported [24]. This finding is reinforced by the observation that “traveller” populations, which have poorer living standards, are at a lower risk of developing CD [25], [26]. A non-random distribution of the disease in sibships with multiple cases suggests that these factors may be shared by the affected sibs during childhood [27]. Finally, because inflammation occurs in the digestive tract, antigens present in the gut lumen including diet components and/or intestinal bacteria are suspected to play a role in CD.

In summary, it can be postulated that CD is linked to one or more familial environmental factor(s) related to an occidental modern way of life, domestic hygiene, diet and infectious agent(s). For this reason, we previously postulated that food refrigeration is a good candidate risk factor for CD [28]. The aim of this study is to understand whether exposure to domestic refrigeration during childhood is associated with CD later in life. We thus conducted a case control study in Iran where the outbreak of the disease is occurring now and by consequence where the exposure of genetically at-risk people is supposed to have been generalised more recently than in Europe or North America.

## Materials and Methods

Patients and controls were recruited in Shariati Hospital and three satellite gastroenterology consultation Clinics in different parts of Tehran. In order to limit recruitment biases associated with health care access, patients and controls were followed by the same gastroenterologists: (RM, SNM, HV and RS). Patients were defined as CD according to recognised diagnosis criteria [29]. In summary, the diagnosis was supported by clinical, radiological, endoscopic and histologic data. CD was defined by segmental inflammatory lesions of the digestive tract. In case of a continuous inflammation of the colon, colonic CD was retained if there was i) no rectal involvement or ii) additional lesion of the digestive tract including perianal lesion or iii) granulomas or iv) strictures or v) transmural inflammation. Controls were recruited among patients followed for irritable bowel syndrome (IBS) according to the Roma 3 classification. Age-matching required age within 5 years of the case.

199 cases and 207 controls were recruited within a 26 months period (July 2004 to August 2006). All the consecutive eligible patients from each clinic were enrolled in the study. Subjects were eligible for inclusion if they were being treated for CD or IBS by an expert gastroenterologist. Reliability of the responses to the questionnaire was validated by reinterviewing a subset of 25 subjects in each group, at least 6 months after the original inquiry. No major differences between the two interviews were detected (data not shown).

All subjects were interviewed by FM who filled the questionnaire. For CD patients, the information on the disease included the date of diagnosis, the location of lesions, the presence of granulomas, fistulas, strictures, extra-intestinal complications, and the therapeutic options. For all participants, the registered items consisted in: date of birth, gender and associated disease, head of family education, head of family's occupation and cigarette smoking. In addition, the questionnaire investigated the exposure to a home refrigerator and freezer at time of the study and in childhood. In case of non exposure since birth, the age of refrigerator/freezer acquisition was noted. Additional items on hygiene and comfort at home were finally recorded.

Continuous variables were described as mean (standard deviation) or median (quartiles) depending on the Gaussian nature or not and qualitative variables as frequency (percentages). Distributions of qualitative variables among patients and controls were compared using the Chi-2 test.

Influence of fridge exposure was studied first by the mean of conditional logistic regression model. Exposure at birth was defined as fridge acquisition at/or before firth birthday. We analysed the age-matched data as initially planned. In this model, cases and controls were matched for their date of birth using stratum of 5 years thus analysis was performed allowing varying number of cases and controls matched. Second, because the date of birth was significantly lower in cases than in controls (respectively 33.5 and 37.1 years, P = 0.002) and because this difference was able to affect the results (considering that exposure to domestic cold has changed within the studied period), we also analyzed the data without age matching. In this new set of analyses, current age was treated as a standard variable using standard logistic model. In addition, all models were adjusted on sex and tobacco use, factors to be known being related to CD. All tests were two-sided and alpha level of 5% was considered as statistically significant. Statistical analyses were performed using the SAS 9.1 software package for PC computer (SAS Inc, Cary, NC).

The study protocol and questionnaire were reviewed and approved by the ethic committee of the digestive disease research center of Tehran University of medical sciences (OHRP number: IRB00001641). All participants gave written informed consent.

## Results

### Description of the studied population

Respectively 97% and 92% of CD and IBS eligible patients accepted the interview. Controls where chosen to have the same age as the patient +/−5 years. The mean age of cases and controls were 34 (Deviation Standard, DS = 12) and 38 (SD = 13), respectively (P = 0.002). There were 128 (63.7%) female in controls and 97 (48.3%) in cases (P<0.001). Tobacco exposure was low and comparable in patients and controls (respectively 10.7% v/s 9.2% of current smokers and 6.6% v/s 5.3% of ex-smokers, NS). Among the reported associated diseases for CD patients, we noted one primary sclerosing cholangitis, one autoimmune hepatitis, one spondyloarthropathy and one breast cancer. Among the IBS group, we found two patients suffering of depression and nine with gastro-eosophageal reflux.

**Figure 1:**
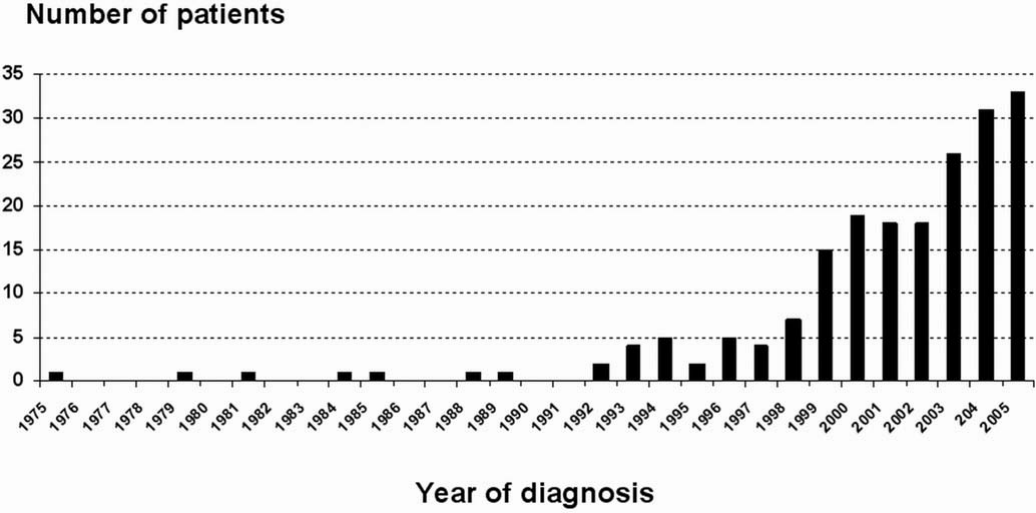
Year of diagnosis for the enrolled CD patients.

**Figure 2:**
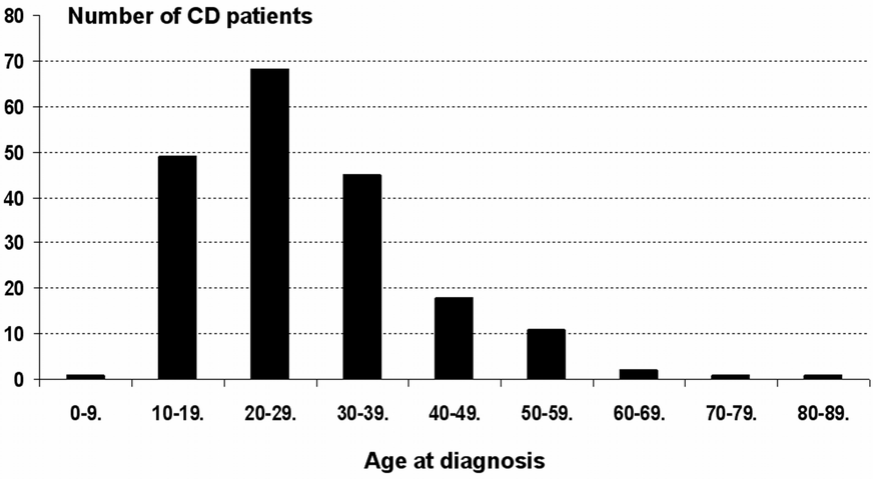
Age-specific incidence of CD in the studied cohort.

In accordance with previously published epidemiological data, most of the diagnoses were made only recently since 90% of CD cases were diagnosed after 2002 (figure 1). In patients, the disease most often occurred in the third decade (mean age of diagnosis 29 years, figure 2). Forty percents of patients had a colonic disease, 21% had small intestinal lesions only, 30% had lesions in colon and small intestine while 9% had a predominant perianal disease. Granulomas were found in 20% of patients. Strictures and fistulas were found in respectively 15% and 14% of patients. Treatments consisted in 5ASA (74%), steroids (72%) immunosupressors (65%) and surgery (12%).

### Exposure to refrigerator at home

**Figure 3:**
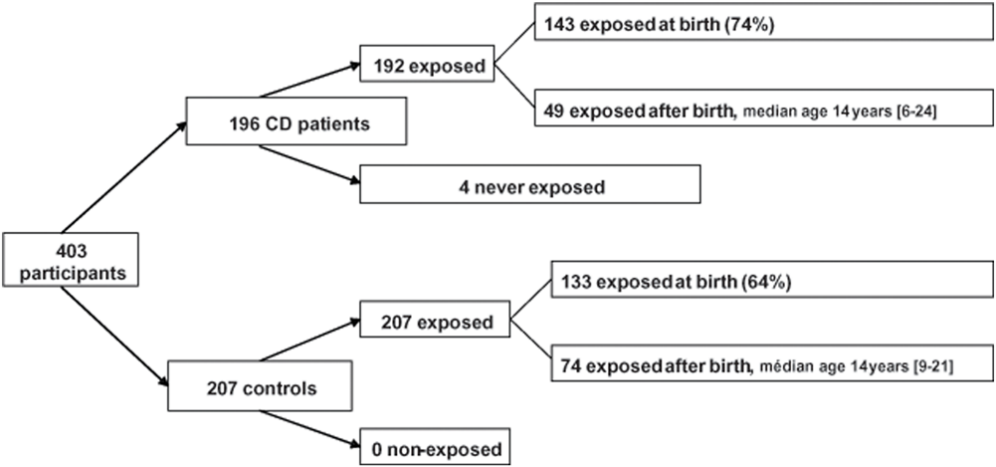
Exposure to refrigerator: flow-chart of the population.

**Figure 4:**
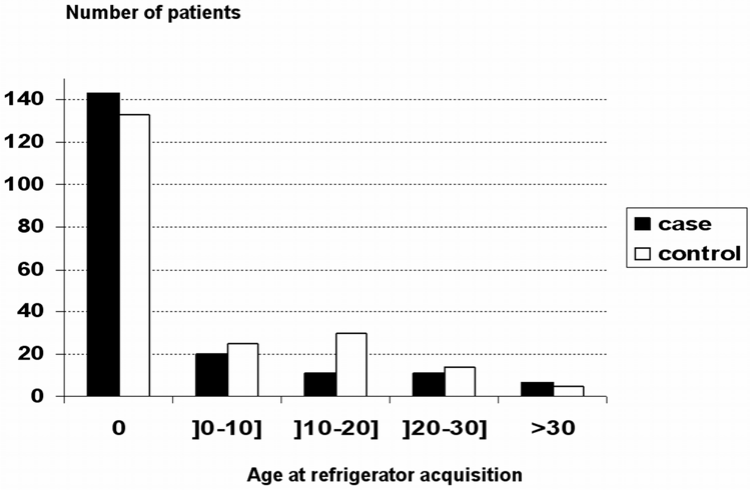
Distribution of the ages of refrigerator acquisition.

Most cases (74%) and controls (64%) were exposed lifetime to domestic refrigeration and the vast majority of patients and controls were refrigerator owners at the time of the interview (figure 3, flow chart). However, patients were exposed earlier than controls to the refrigerator (X2 = 9.6, df = 4, P = 0.05, figure 4).

The conditional logistic model on age-matched data found that exposure to a refrigerator at birth was higher in cases than in controls but this difference did not reach significance (exposure at birth, OR = 1.87 (0.88–3.96); p = 0.10). Because age-matching was weak, we analysed the data without age-matching but with adjustment on current age. We thus found that the association reached significance (OR = 2.08 (1.01–4.29); P = 0.05).

### Other variables

Several variables were recorded in order to better understand which co-factors were associated with the exposure to domestic refrigeration in childhood. In patients, the percentages of head's family with none, elementary school, high school and college graduations were 16%, 25%, 28% and 31% while these percentages were respectively 19%, 39%, 24% and 17% in controls. No differences were seen with regard to the living in rural/urban area, the presence of pets at home, the size of sibships or the number of children sharing the same bedroom (table 1). In addition to domestic refrigeration, additional items present at home and reflecting good standards of comfort and hygiene were also associated with CD including television, car and washing machine (table 2). Interestingly, we obtained results comparable with those seen for the refrigerator when looking at the exposure to freezer at home. Indeed 65% of patients and 50% of controls were exposed since birth and patients were exposed earlier than controls (Χ^2^ = 17.1, df = 4, P = 0.002).

**Table 1:** Domestic hygiene global parameters during childhood*.

Parameter	Crohn's Disease n = 196 (%)	Controls n = 207 (%)
Urban/rural	89%/11%	85%/15%
Pets	69%	70%
Nb of siblings	5 (4–7)*	6 (5–7)**
Nb of sibs in the bedroom	2 (2–4)*	2 (2–4)**

Childhood is defined by exposure before the 16th birthday. ** Median (1st-3th quartile).

**Table 2:** Comfort at home in CD patients and controls.

Equipment	Ownership at birth CD (n = 196)	Ownership at birth controls (n = 207)	Delay before exposure CD *	Delay before exposure controls*
Refrigerator	143 (74%)	133 (64%)	14 [6–24]	14 [9–21]
Freezer	115 (64%)	101 (50%)	16 [7–24]	18 [12–28]
Running water	142 (72%)	148 (71%)	12 [6–18]	14 [9–20]
WC	142 (72%)	148 (71%)	18 [10–25]	19 [9–24]
Hot tap water	123 (63%)	122 (59%)	16 [7–24]	16 [10–21]
Separate bathroom	121 (62%)	118 (57%)	17 [9–25]	19 [10–26]
Television	131 (67%)	115 (56%)	16 [9–24]	14 [8–20]
Washing machine	102 (52%)	75 (36%)	15 [8–24]	19 [11–27]
Car	88 (45%)	64 (31%)	21 [7–27]	19 [9–27]
Central heating	36 (18%)	29 (14%)	19 [11–27]	26 [15–33]
Computer	0 (0%)	2 (1%)	21 [16–33]	32 [19–40]
Micro-wave owen	1 (0.5%)	0 (0%)	26 [20–37]	31 [21–41]

* Data are expressed in medians and quartiles.

## Discussion

Even if the long-term trend of CD demonstrates the role of the environment, epidemiological studies have failed to identify the causative risk factor(s). Usually, these studies compare retrospectively the exposure to specific factors in healthy controls and CD cases. However, more common is the risk factor in the general population, less frequent are the non-exposed healthy controls and less powerfull are the case-control studies. Thus, a widespread environmental factor could not be easily detectable. This unfavorable situation is expected in European and North American countries which are characterised by a standardised way of life and a stabilised incidence of the disease. At the opposite, in a country where CD is an emerging public health problem, it can be postulated that the exposure to a putative CD risk factor is not ubiquitous in the general population. The association between the risk factor and the disease may thus be easier to detect.

In Iran the first reports of ulcerative colitis appeared 22 years ago and the first two studies emphasized that CD was not existent [30]–[32]. The first CD case was reported 7 years ago and now CD is as common as ulcerative colitis [33], [34]. Thus in Iran as in Western countries, UC appeared first, followed by CD 15–20 years later and today CD is an emergent public health problem.

The clinical presentation of CD in Iran appears consistent with the classic presentation of the disease. The age-specific incidence curve is in agreement with many other epidemiological data [7], [34]. The location of CD lesions is also consistant with previous reports on other CD cohorts [35]. The proportion of patients with epithelioid granulomas is relatively low when compared to other series but this finding may be explained by the limited follow-up and the limited number of surgical interventions, two factors known to affect the percentage of granulomas detected in CD patients [36]. Finally, the percentages of complications are compatible with the literature [37]. Altogether, these observations suggest that CD clinical features in Iran are similar to those previously reported in other countries [34], [38]

In order to test the hypothesis of an increased exposure of CD patients to refrigerator at home, we compared CD patients and controls. As controls, we choose IBS patients followed by the same gastroenterologists. This strategy was drawn in order to limit the differences between groups in terms of area of residence, access to health care, socio-economic status, and putative other uncontroled biases. The diagnosis of IBS was established by expert gastroenterologists and was based on classic Roma III criteria excluding the possibility that the control group would contain a significant number of misclassified CD patients. The list of associated diseases and the sex ratio observed in this control group, confirmed that it could be seen as acceptable. It can also be questioned if these controls reflect the general Iranian population. Data from the literature indicate that IBS may be associated with a high socioeconomic status [39]. By consequence, the results presented here should be interpreted with caution.

We report here an increased exposure to refrigerator at birth in the patient group. It must be acknowledged that the two strategies of statistical analyses modelled differently current age of patients and controls. In the standard logistic model, age was included as single linear term whereas the matched analysis did not depend on this assumption, possibly explaining differences reported in results and statistical significance. Data on the exposure to freezer confirm the association between CD and domestic refrigeration. However, as expected, exposure to a family domestic refrigerator is not an isolated factor. Indeed, it reflects better standards of domestic hygiene and comfort in general and a higher level of education. It is thus impossible to exclude that refrigeration at home is a confounding factor even if, among the domestic equipments reflecting domestic hygiene and comfort analysed in this study, refrigeration can be seen as one of the best candidates when one considers both the strength of the association and the putative mechanisms of disease (table 2).

Our conclusion is in accordance with a previous publication by Forbes A. and Kalantzis T. [40]. These authors have conducted a retrospective case-control study in a cohort of inflammatory bowel disease patients from United Kingdom. In the older patients, they found a 4.7 year difference between CD patients and controls for the age at first fridge.

These two studies support two different reported hypotheses for CD. In both cases, the theories predict that the exposure to the refrigeration at home is equal or higher in the group of patients when compared to a control group from the general population. The first one, known as the hygiene hypothesis postulates that a more sanitary childhood environment should prevent the host from developing tolerance to organisms that may present later in life, with the subsequent development of CD lesions [41]. The other one is known as the cold chain hypothesis discussed above [28].

At the opposite of the hygiene hypothesis, the cold chain hypothesis predict that exposure to refrigerated food is directly correlated with disease occurrence in genetically at risk people. Obviously, the presence of a fridge at home does not resume the exposure to refrigeration. The cold chain is complex and most of the food eaten today has been conserved at low temperature at some time. Exposure to domestic refrigeration is thus only a rough estimate of the exposure to refrigerated food and more sophisticated studies would be required to further analyse this risk factor. For example, it is highly probable that CD patients who do not have a refrigerator today (as it is the case for 4 patients in this study) eat refrigerated food. By consequence, it was out of the scope of this case-control study to demonstrate a causal relationship between refrigeration and CD which would require experimental interventions in studied populations.

The cold chain hypothesis assumes that CD results of the chronic exposure to bacteria able to grow at low temperature and known as psychrotrophic bacteria. Among these bacteria, *Yersinia* species may be seen as the best candidates for several reasons. Firstly, many authors reported cases where CD developed after a documented infection by *Yersinia* species. More recently, a Scandivanian group confirmed these individual reports at the population level, showing that CD is more frequent in the years following a documented *Yersinia* infection [42]. Secondly, mesenteric lymph node mononuclear cells of CD patients have been shown to have an increased proliferative response toward Yersinia antigens [43]. Thirdly, at least two groups have independently found Yersinia species in CD lesions [44], [45]. Finally, we have recently shown that mice invalidated for CARD15/NOD2 are characterised by an abnormal response after oral infection by Yersinia pseudotuberculosis [46]. Altogether, these data support the cold chain hypothesis and suggest pursuing investigations.
